# Rapid Body-Wide Transcriptomic Turnover During Rhesus Macaque Perinatal Development

**DOI:** 10.3389/fphys.2021.690540

**Published:** 2021-06-10

**Authors:** Wenqian Zhang, Wei Wang, Manman Zhao, Christoph W. Turck, Ying Zhu, Guang-Zhong Wang

**Affiliations:** ^1^School of Life Sciences, Guizhou Normal University, Guiyang, China; ^2^CAS Key Laboratory of Computational Biology, Shanghai Institute of Nutrition and Health, University of Chinese Academy of Sciences, Chinese Academy of Sciences, Shanghai, China; ^3^State Key Laboratory of Medical Neurobiology, MOE Frontiers Center for Brain Science, Institutes of Brain Science, Fudan University, Shanghai, China; ^4^Max Planck Institute of Psychiatry, Proteomics and Biomarkers, Munich, Germany

**Keywords:** brain development, peak regulation, perinatal development, macaque, developmental pattern

## Abstract

An hourglass cup-shape pattern of regulation at the molecular level was detected during the development of the primate brain. Specifically, a peak of temporally differentially expressed genes around the time of birth has been observed in the human brain. However, to what extend this peak of regulation exists among species has not been investigated in great detail. Here, by integrating multiple large-scale transcriptome data from rhesus macaques, we confirmed that a similar differential expression peak exists during the development of the macaque brain. We also found that a similar peak exists during the development of other organs, such as liver, testis, kidney and heart. Furthermore, we found that distinct pathways are regulated in the peak period of those organs. Our results highlight the importance of co-evolution of diverse organs during critical periods of perinatal development in primates.

## Background

The brain is a very complex structure that consists of a large number of inter-connected neurons (Miller and Cohen, 2001; Herculano-Houzel, 2009; Silbereis et al., 2016; Cheng and Haorah, 2019), and its formation requires precise orchestration of gene expression. Despite decades of efforts, the transcriptional dynamics of different brain regions across development has still not been fully elucidated. Our understanding of primate brain development is further limited by accessibility and ethical concerns. To understand the developmental dynamics of the primate nerves system, large-scale sequencing has been employed to analyze and compare different brain regions during human and other primate brain development (Wang et al., 2009; Costa et al., 2010; Pletikos et al., 2014; Wu et al., 2015; Lukowski et al., 2019; Wang and Wang, 2019b).

The development of the human nervous system typically takes several years to decades. At the molecular level, the precise regulation of gene expression is critical for its development and function, with significant implications for molecular mechanisms implicated in mental and neurological disorders. When comparing the differences between the developing human and its closely related non-human primate brains (Liu et al., 2012; Miller et al., 2014; Zhu et al., 2014, 2018; Li and Wang, 2019), it was proposed that a cup-shaped or hourglass pattern of inter-species or inter-regional differential expression exists at the transcriptome level, either in humans or in rhesus monkeys. Transcriptome regulation patterns in the human brain regions confirm a two-peak’s regulation during human brain development (Wang and Wang, 2019a). The first peak occurs around perinatal periods, and may be involved in morphine addiction, cell proliferation, growth, and migration paths. The second peak occurs in childhood, and is primarily involved in dopamine-enabled synapses, learning and memory.

The rhesus macaque (*Macaca mulatta*) is the most widely investigated non-human primate. Its transcriptome provides a unique opportunity to study the developmental characteristics in non-human primates. Here, by an integrative analysis of macaque transcriptome, we examined whether the early peak regulation detected in the human brain also exists in other primates, and more importantly, whether this early peak can be extended to the development of other parts of the body (Napier, 1967; Chen and Li, 2001; Cáceres et al., 2003; Leigh, 2004; Rilling, 2006; Liu et al., 2012; He et al., 2017). Answers to these questions provide the basis for our exploration of the mechanisms of rhesus monkey development and help us to better understand the differences between the development of human and non-human primates.

## Results

### Dynamics of Rhesus Macaque Brain Transcriptomes Across Early Developmental Periods

**FIGURE 1:**
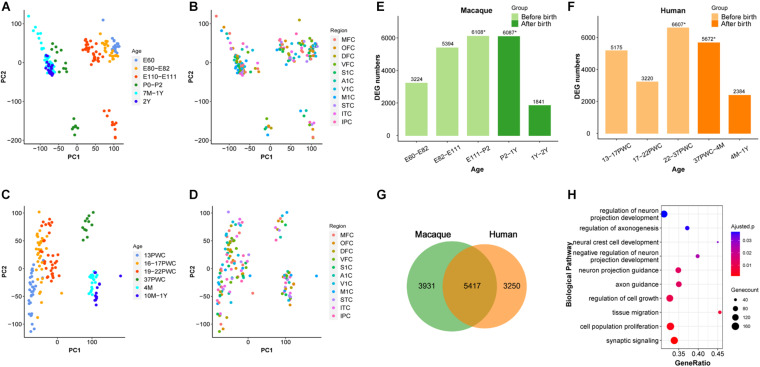
Similarity between early developmental transcriptome of macaque and human brains. **(A,B)** PCA plot of 179 rhesus macaque brain samples, with each dot representing a sample. **(C,D)** PCA plot of 147 human brain samples, with each dot representing a sample. **(A,C)** The developmental period was used to mark the samples. **(B,D)** The different brain regions were used to mark the samples. **(E)** Number of differentially expressed genes (DEG) in rhesus macaques detected at adjacent time periods, the *x*-axis represents the time period, the *y*-axis is the number of DEGs. **(F)** Number of DEGs in human detected at adjacent time periods, the *x*-axis represents the time period, the *y*-axis is the number of DEGs, * denotes the peak, and the different colors represent the segmentation before and after birth. **(G)** The number of DEGs overlapping at peak period between human and rhesus macaques. **(H)** GO enrichment analysis of overlapping DEGs, sorted from smallest to largest by *p*-value. Smaller *p*-values are indicated by darker red color. The size of the circle represents the number of DEGs and *x*-axis represents the odd ratio.

We firstly analyzed 179 macaque brain transcriptome samples (Zhu et al., 2018), ranging from early embryonic stage to 2-year old, and covering 11 neocortical areas: medial prefrontal cortex (MFC); orbital prefrontal cortex (OFC); dorsolateral prefrontal cortex (DFC); ventrolateral prefrontal cortex (VFC); primary motor cortex (M1C); primary somatosensory cortex (S1C); primary auditory cortex (A1C); primary visual cortex (V1C); superior temporal cortex (STC); inferior temporal cortex (ITC); and inferior posterior parietal cortex (IPC). Principal component analysis (PCA) was used to explore the global relationships among these samples (Figures 1A,B). The first principal component (PC1) explained the majority of the variations in gene expression by separating the samples by developmental periods (early to late). However, we found no obvious clustering of samples by neocortical areas (Figure 1B). Similar results were observed using 147 samples of the same 11 human neocortical areas from similar developmental periods (Figures 1C,D). These results are consistent with previous reports (Kang et al., 2011) and suggest that the developmental macaque neocortex shares a highly similar transcriptome program with that of the human neocortex and the developmental period is a major factor affecting transcriptome divergence during primate development.

### A Transcriptional Regulation Peak Around Birth During Early Brain Development in Rhesus Macaque

It was shown previously that early development of the human brain is highly dynamic and there is a peak of temporal differential gene expression around birth (Kang et al., 2011). We firstly validated if the transcriptional regulation peak exists in rhesus macaques by using RNA-sequencing data (Zhu et al., 2018). As shown in Figure 1, indeed there was a dramatic increase of differentially expressed genes (DEGs) during prenatal development, and a big decrease during postnatal development in macaque, forming a peak around birth (E111-P2 and P2-1Y) (Figure 1E and Supplementary Table 1). Additionally, we further validated that this pattern is conserved in the human neocortex, and the number of DEGs was similar between macaque and human neocortices around birth (22-37PWC and 37PWC-4M) (Figures 1E,F and Supplementary Figure 1). In total, we found 5,417 differentially expressed genes at this early peak period of brain development are shared between the two species, which accounts for 58% of the total DEGs during the early development of rhesus macaque neocortex (Figure 1G and Supplementary Figure 2). The large overlap indicates that the critical developmental peak periods between the human and rhesus macaque neocortices are to a large extent conserved. The functional enrichment of these genes reveals that they are related to biological processes such as neural cell development (Benjamini-Hochberg correction for *P* = 1.08e-3), regulation of axonogenesis (Benjamini-Hochberg correction for *P* = 1.36e-3), axon guidance (Benjamini-Hochberg correction for *P* = 3.57e-4), and synaptic signaling (Benjamini-Hochberg correction for *P* = 2.69e-5) (Figure 1H).

**FIGURE 2:**
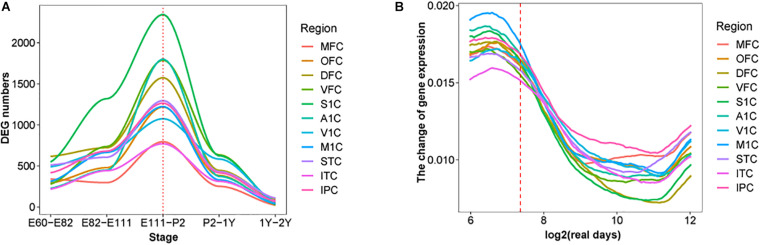
DEGs in each brain region over time in macaque brain. **(A)** The distribution of DEGs in 11 brain regions in rhesus macaques, the *x*-axis represents developmental period, the *y*-axis is the number of DEGs, colors represent distinct brain regions and the red dotted line indicates birth time. **(B)** Changes in fitting curves of different brain regions in rhesus macaques, the expression value of each gene was fitted over time, 100 time points were selected and then the top 20% of the greatest difference was plotted.

Moreover, the early transcriptional regulation peak was observed in each of the 11 macaque neocortical areas with a similar number of differentially regulated genes (Figure 2A). In contrast, estimating the change rate of gene expression by interpolating and approximating expression on evenly spaced points based on a Gaussian process model, reveals a high change rate during prenatal development and a sharp decline at the late fetal period. Different temporal patterns of DEGs and change rate indicates that a small group of DEGs with high change rate accounts for dynamics in early prenatal development, while a larger group of DEGs with low change rate contributes to temporal differences around birth (Figure 2B). Overall, these results illustrate the co-evolution of different neocortical areas during development in both macaque and human. We next examined whether this pattern can be extended to other brain regions and body organs.

### Transcriptional Regulation Peak During Early Development in Other Rhesus Macaque Organs

**FIGURE 3:**
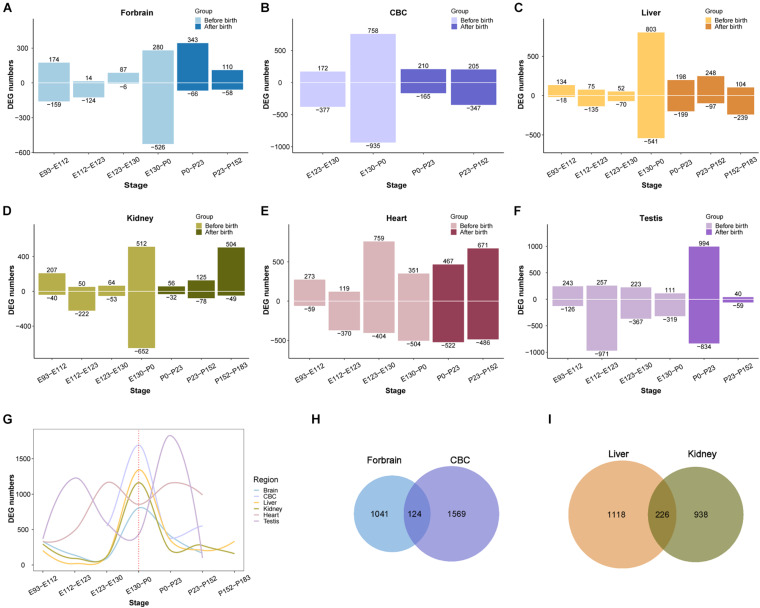
Organ DEG distribution in rhesus macaques. **(A–F)** Show the distribution of DEGs across development in forebrain, cerebellum (CBC), liver, kidney, heart, and testis. The *x*-axis shows age, the *y*-axis shows the number of DEGs in each period. Positive and negative indicate the number of upregulated and downregulated DEGs, respectively. **(G)** The distribution of overall DEGs across development in the 6 organs, the first regulatory peak starts mostly before birth. **(H)** The overlap of DEGs between ForeBrain and cerebellum (CBC). **(I)** overlap of DEGs between liver and kidney.

After demonstrating that the early transcriptional regulation peak also exists in the development of the rhesus macaque brain, we next wondered if this pattern can be extended beyond the development of the central nervous system. We performed a similar differential expression analysis of the transcriptome of other macaque organs (Cardoso-Moreira et al., 2019). For this purpose we collected developmental RNA-seq data from rhesus macaque forebrain (E93-P152, 16 samples), cerebellum (E123-P152, nine samples), kidney (E93-P183, 17 samples), liver (E93-P183, 18 samples), heart (E93-P152, 14 samples), and testes (E108-P152, 16 samples) (Cardoso-Moreira et al., 2019). We found that the regulation of the transcriptome reached a peak around birth time not only in the forebrain, but also in the cerebellum, kidney, liver, and testis. However, the peak was earlier in macaque heart than in other organs (Figure 3). This suggests that early transcriptional regulation peak exists in many macaque organs, and in most organs developmental changes occur prior to birth.

Additionally, we observed that the number of differentially expressed genes detected in those organs ranges from 1,100 to 1,800, with many overlaps between each other (Supplementary Table 2). For instance, there are 124 differentially expressed genes overlapping between forebrain and cerebellum, and 226 DEGs overlapping between liver and kidney, which are both more than expected by chance (OR = 1.95 and OR = 5.64, *P* = 2.465e-10 and 2.2e-16, respectively, Fisher’s exact test). The overlapped DEGs indicate those genes play important roles during the development of multiple organs, which may involve in more fundamental pathways than other genes in the tissue. Together all above results implicate the emergence of tissue specific function during the regulation of this peak in early development.

### Functional Analysis of Transcriptional Regulation Peak of Early Organ Development

**FIGURE 4:**
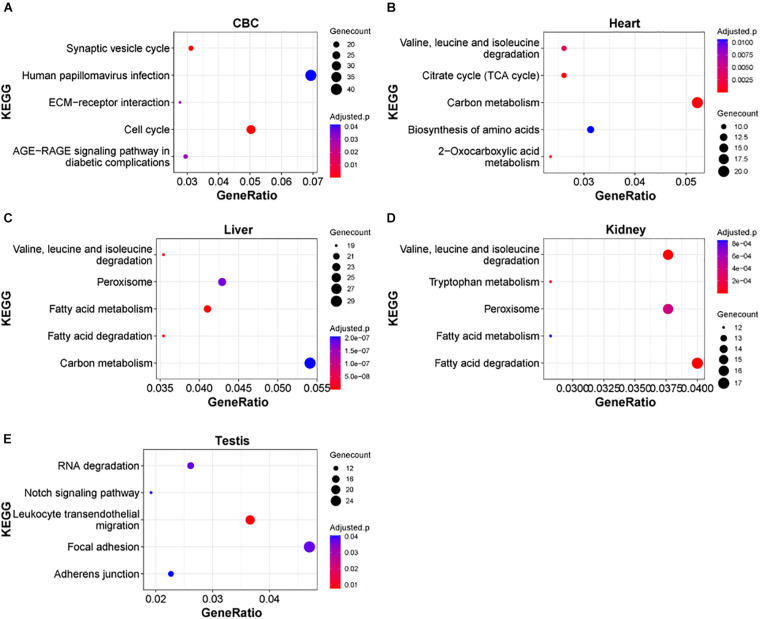
KEGG pathways of DEGs in rhesus macaque organs. **(A–E)** Represent top enriched functional categories in the developmental DEGs of cerebellum (CBC), heart, liver, kidney, and testis. Pathway terms were sorted from smallest to largest by *p*-value. Smaller *p*-values are indicated by darker red color. The size of the circle represents the number DEGs enriched and the *x*-axis represents the odd ratio.

Next, we examined the functional significance of the transcriptional regulation peak across multiple macaque organs. It seems that the functional enrichment of this peak is quite diverse (Figure 4). For instance, the top enriched pathways in heart are *2-Oxocarboxylic acid metabolism* (adjusted *P* = 1.17e-5), *Carbon metabolism* (adjusted *P* = 1.64e-4) and *Citrate cycle (TCA cycle)* (adjusted *P* = 1.64e-4), while the top enriched pathways in liver are *Fatty acid metabolism/degradation* (adjusted *P* = 1.81e-10 and 4.67e-10). *Valine, leucine and isoleucine degradation* (adjusted *P* = 1.55e-8), and *Peroxisome* (adjusted *P* = 1.77e-7) and in testis the top enriched pathways are *Notch signaling pathway* (adjusted *P* = 0.0406), *Adherens junction* (adjusted *P* = 0.0406), and *Tight junction* (adjusted *P* = 0.0406). The results suggest that distinct sets of DEGs are important for the functional differentiation of different organs during early development. In addition, we found that in different parts of the brain, *Axon guidance*, *GABAergic synapse*, *Wnt signaling pathway*, and *Circadian entrainment*, were the major pathways enriched in this peak process (Supplementary Figure 4), suggesting that these common pathways play an important role in the functional differentiation of the brain.

## Discussion

In this study, through an integrative analysis of macaque organ early development, we observed that similar to the human brain, the developmental period is an important factor influencing gene expression patterns across brain regions of the macaque brain. We also found a transcriptional regulation peak at the molecular level during early macaque brain development, by performing temporal differential expression analysis of transcriptomes from multiple brain regions across several years. Additionally, we demonstrated that this peak could be extended to other macaque organs, which occurs at a similar developmental period as the brain. This body-wide transcriptome turnover involves different cellular pathways. We submit that this transcriptome wide regulatory peak is important for the functional differentiation of each organ.

The extensive transcriptome turnover may have important developmental implications. Firstly, it suggests that similar regulatory programs driving the molecular dynamics of the transcriptome may be involved during this critical period in different organs. Secondly, it supports the hypothesis that a similar level of selective constraints exists at a particular developmental period in different organs. Lastly, it suggests that different cell types may emerge at this early developmental period, which can be further classified by single cell sequencing technologies (Zhong et al., 2018; Potter, 2018).

Can this regulation peak be extended to other species? Very possibly. Previously it was shown that the gene expression across multiple vertebrate species share similar patterns and purifying selection is decreased during development (Cardoso-Moreira et al., 2019). At least for human and other primate species, the early peak regulation across organs might be a conserved feature. For the brain, recently evolved genes are supposed to play a role in the early development, and provide functional innovation (Laurent and Dominique, 2000; Stern, 2000; Carroll, 2005; Carolin et al., 2008; Kaessmann, 2010; Zhang et al., 2011). Large-scale and high sampling density datasets will be required for this type of investigations.

Finally, the early transcriptional regulation peak may be closely linked to tissue specificity, by promoting functional diversity. For instance, in the brain, multiple brain regions have reached the developmental peaks at roughly the same time (Wang and Wang, 2019a), which indicates that the fetus undergoes significant developmental changes before birth. In terms of the differences of this peak between macaque and human, our data suggest that macaque has a greater number of DEGs before birth while humans have greater number of DEGs after birth, at least in the brain. Beyond that, integrative analyses of single-cell transcriptome data would provide the insight of the contribution of diverse cell types during this critical developmental period. Thus, high quality single-cell datasets are desired for the early developmental of both macaque and human. In all, a complete understanding of the physiological significance of the differences at multiple levels will be important for the field of developmental biology.

## Materials and Methods

### Data Source

RNA-sequencing data for rhesus macaque developmental transcriptome were obtained from our previous study (Zhu et al., 2018), 11 neocortical areas, medial prefrontal cortex (MFC); orbital prefrontal cortex (OFC); dorsolateral prefrontal cortex (DFC); ventrolateral prefrontal cortex (VFC); primary motor cortex (M1C); primary somatosensory cortex (S1C); primary auditory cortex (A1C); primary visual cortex (V1C); superior temporal cortex (STC); inferior temporal cortex (ITC); and inferior posterior parietal cortex (IPC), covering 179 samples were included in the analysis. To examine if macaque and human share high early developmental similarity, 147 human samples from similar developmental periods from the same 11 neocortical areas were collected as well. In total, the transcription profiles from 27,932 genes were used from both rhesus macaque (six developmental periods) and human (six developmental periods) samples. For rhesus macaque early developmental transcriptome analysis, RNA-seq data from 90 samples from 6 organs (forebrain, 16 samples; cerebellum (CBC), 9 samples; kidney, 17 samples; liver, 18 samples; heart, 14 samples; and testis, 16 samples) were used (Cardoso-Moreira et al., 2019).

### Identification of Differentially Expressed Genes During Early Development

To identify differentially expressed genes during development, different developmental periods were first classified. Six age groups were used for subsequent analysis (Zhu et al., 2018). Rhesus macaque age groups (periods): E60, E80-E82, E110-E111, P0-P2, 7M-1Y, 2Y. Human age groups: 13PWC, 16-17PWC, 19-22PWC, 37PWC, 4M, 10m-1Y. Considering the possible difference between neocortical areas, the 11 neocortical areas were analyzed separately. Differential expression analysis of different neocortical areas was conducted as follows: (1) One-way analysis of variance (ANOVA) was used to identify DEGs in each neocortical area using age as a variable factor (Chambers and Hastie, 1992). In order to control for the rate of false discovery (FDR), the significant level (P) from the ANOVA test was corrected using the Benjamini-Hochberg procedure, and genes with FDR < 0.01 were considered significantly differentially expressed. (2) The remaining DEGs were screened with Tukey’s HSD test to calculate the differences between the two adjacent developmental periods in each neocortical area. DEGs with Tukey’s HSD test between two adjacent periods *P* < 0.01 were selected (Miller, 1981; Grego and John, 1997).

The overall differential expression analysis of the 11 neocortical areas was conducted as follows: (1) we performed ANOVA with age and neocortical area as the main factors. In order to control for the rate of false discovery (FDR), the significant level (P) from the ANOVA test on the age factor was corrected using the Benjamini-Hochberg procedure, and genes with FDR < 0.01 were considered temporally differentially expressed. The DEGs were then screened for Tukey’s HSD test and only age in the ANOVA results was used as a variable factor. Genes Tukey’s HSD test between two adjacent periods *P* < 0.01 were defined as temporal DEGs.

### Functional Gene Enrichment Analysis

After identifying the differentially expressed genes that changed over time, we performed enrichment analyses of the biological functions that might be involved in regulation. Here, we used two methods of function enrichment: Gene Ontology Resource (Ashburner et al., 2000; Carbon et al., 2021) and KEGG enrichment analysis by ClusterProfiler (Yu et al., 2012). The *P*-value was corrected by the Benjamini-Hochberg method and pathways with adjusted *P* < 0.05 were regarded as enriched. The reference set for rhesus macaques in the ClusterProfiler package was used by default^1^.

### Fitting the Gene Expression Trajectory Across Development

To estimate the change rate of expression, we fitted a Gaussian process model for each gene in each brain region to model the gene’s expression trajectory during development, using the R package Tempshift (Zhu et al., 2018). The expression value of each gene at 100 evenly spaced timepoints were calculated, and the differences between adjacent timepoints were then computed as the expression change rate. For all computations, the R-3.6.1 analysis software was used and all codes are available upon request.

## Data Availability Statement

The original contributions presented in the study are included in the article/Supplementary Material, further inquiries can be directed to the corresponding author/s.

## Author Contributions

G-ZW and YZ designed and supervised the project. WZ, WW, and MZ performed the bioinformatics analyses. WZ, G-ZW, CT, and YZ wrote the manuscript. All authors agreed on the final version of the manuscript.

## Conflict of Interest

The authors declare that the research was conducted in the absence of any commercial or financial relationships that could be construed as a potential conflict of interest.

## Footnotes

1 org.mmu.eg.db

## Supplementary Material

Supplementary Figure 1 Upset plot of DEGs from the early brain development of macaque and human.

Supplementary Figure 2 Volcano plot of DEGs from the early brain development of macaque and human.

Supplementary Figure 3 Upset plot of organ-related developmental Dges.

Supplementary Figure 4 Kegg pathways of DEGs in different brain regions in rhesus macaques. **(A–I)** represent top enriched functional categories in the developmental DEGs of Mfc, Dfc, Ofc, Vfc, A1C, S1C, Ipc, Itc, and Stc. Pathway terms were sorted from smallest to largest by *p-value*. Smaller *p-values* are indicated by darker red color. The size of the circle represents the number DEGs enriched and the *x-axis* represents the odd ratio.

Supplementary Table 1 Detailed information of DEGs in 11 brain regions of early developmental peak of rhesus macaques.

Supplementary Table 2 Detailed information of DEGs in six organs of early developmental peak of rhesus macaques.
