# Supplementary figures and images for: Rapid Body-Wide Transcriptomic Turnover During Rhesus Macaque Perinatal Development
Source: Front Physiol. 2021 Jun 10;12:690540. doi: 10.3389/fphys.2021.690540 (PMC8223001; doi:10.3389/fphys.2021.690540)

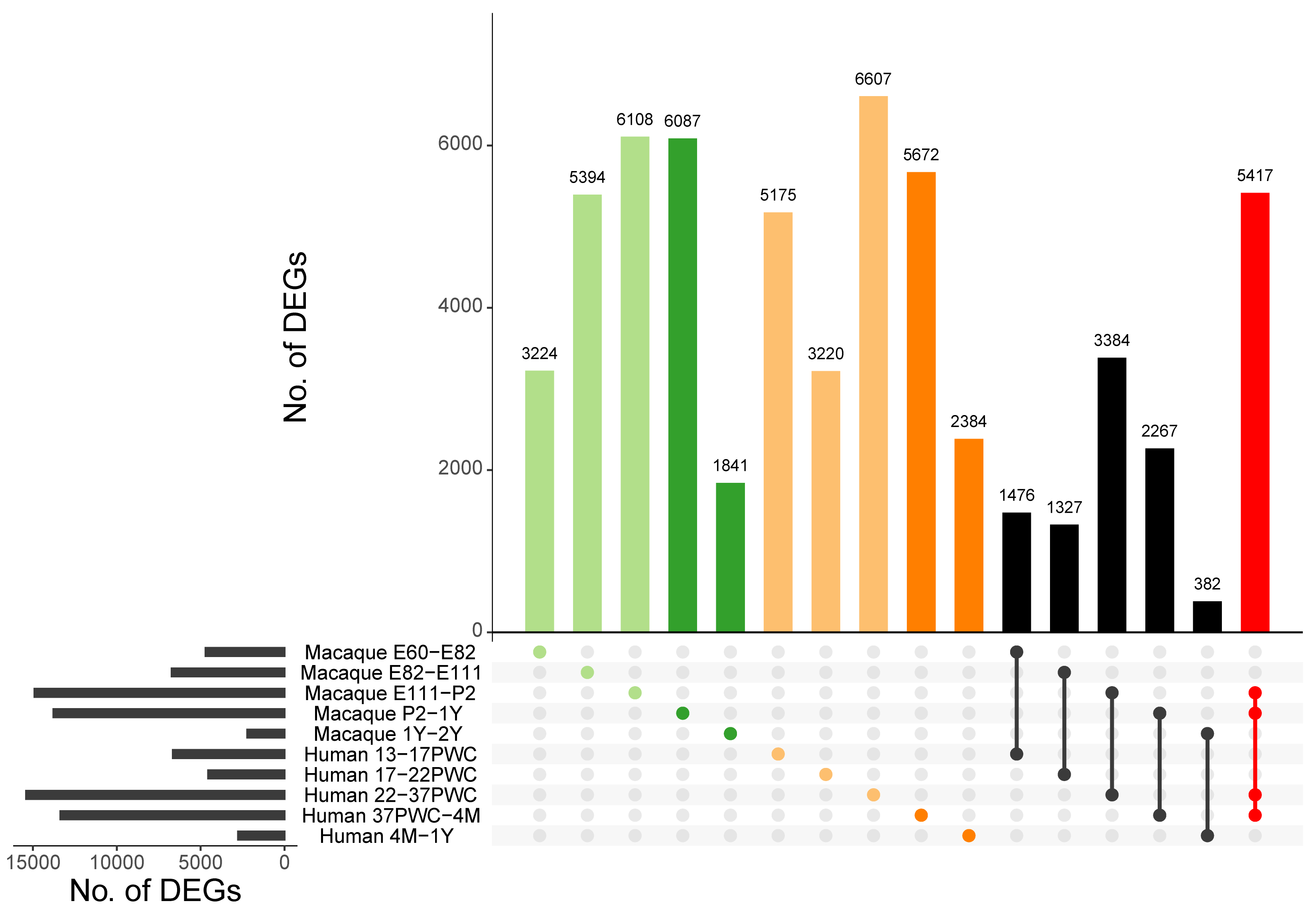

Supplement: Supplementary Figure 1 — Upset plot of DEGs from the early brain development of macaque and human. [file Image_1.TIF]

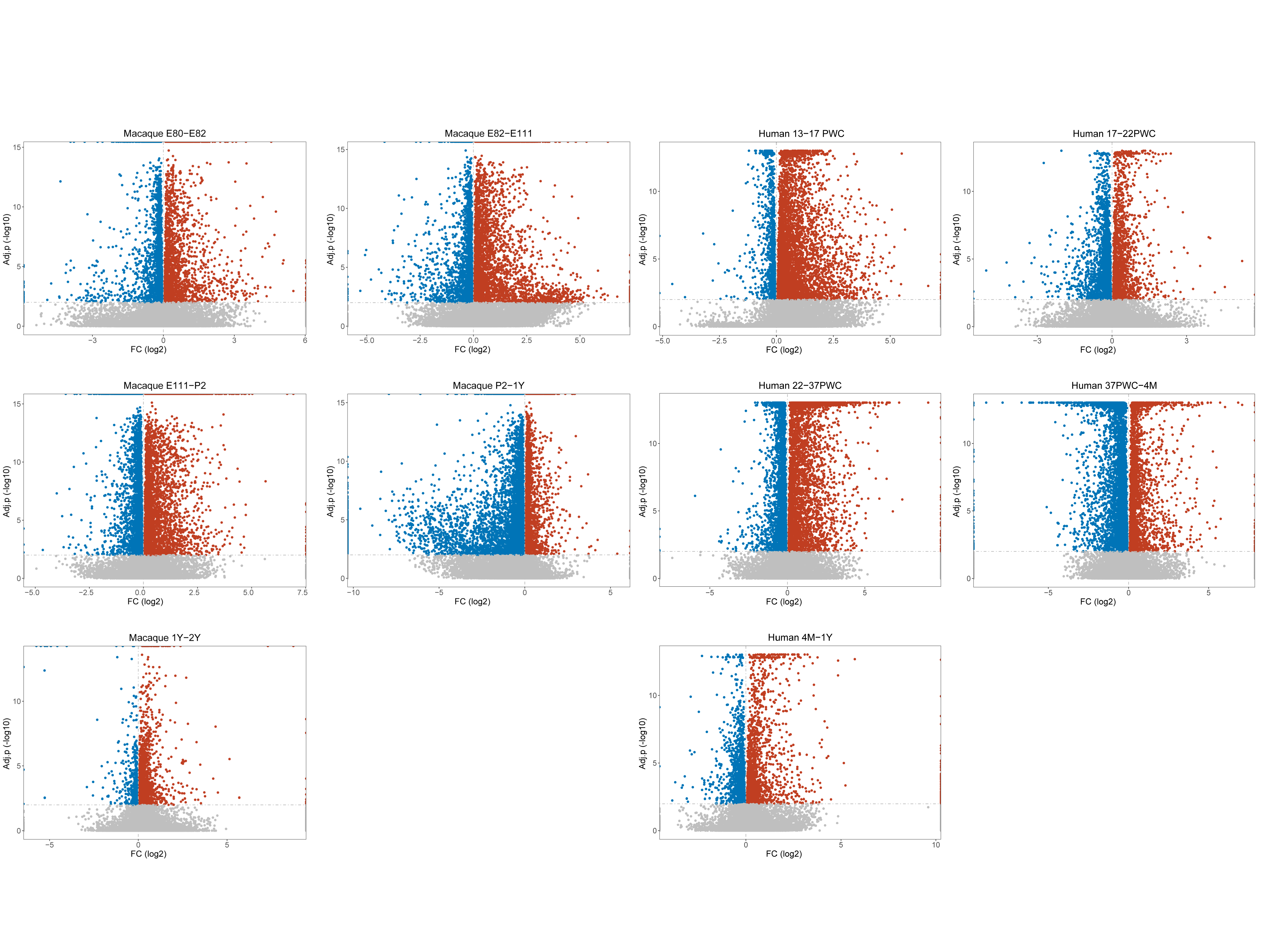

Supplement: Supplementary Figure 2 — Volcano plot of DEGs from the early brain development of macaque and human. [file Image_2.TIFF]

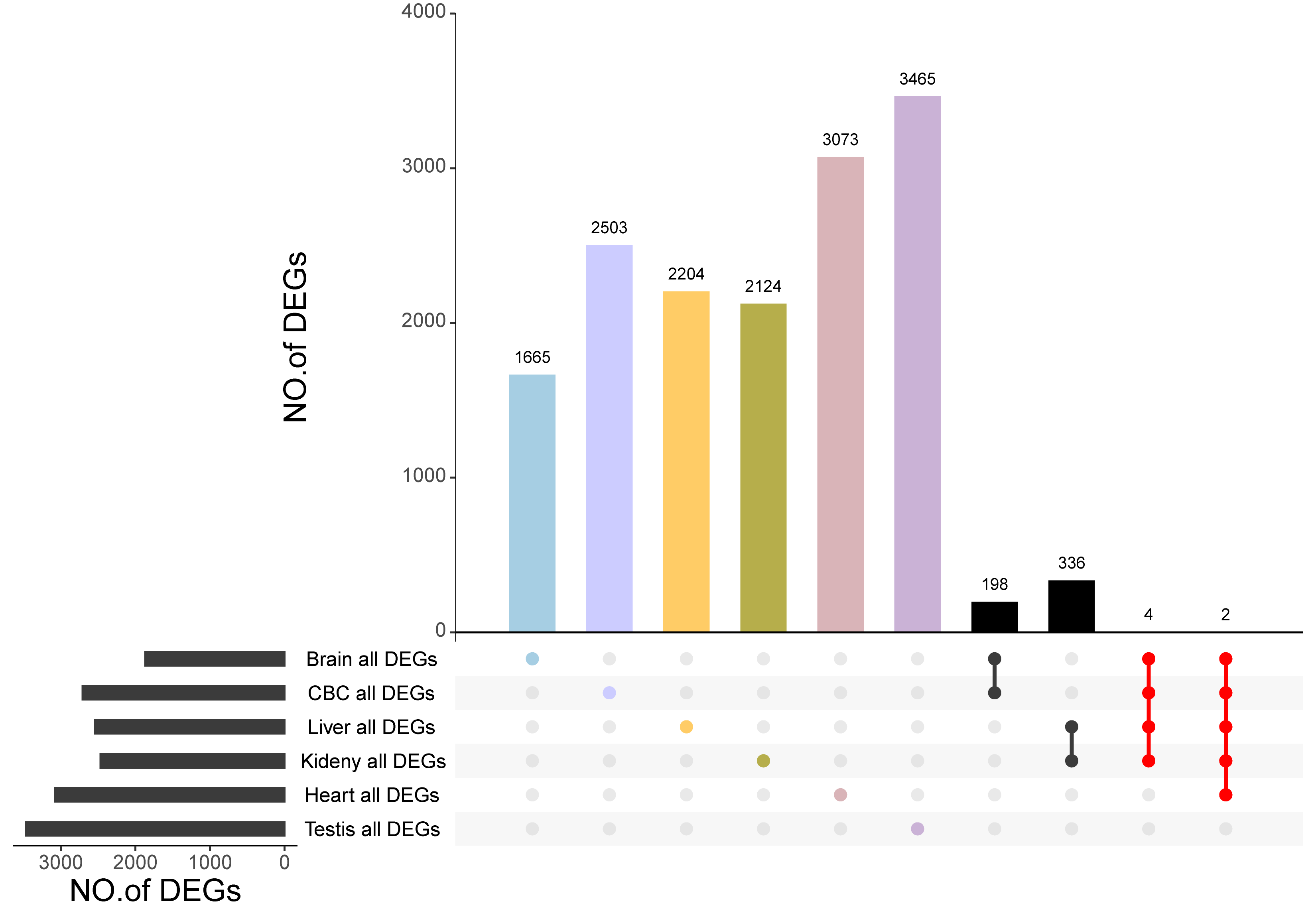

Supplement: Supplementary Figure 3 — Upset plot of organ-related developmental Dges. [file Image_3.TIF]

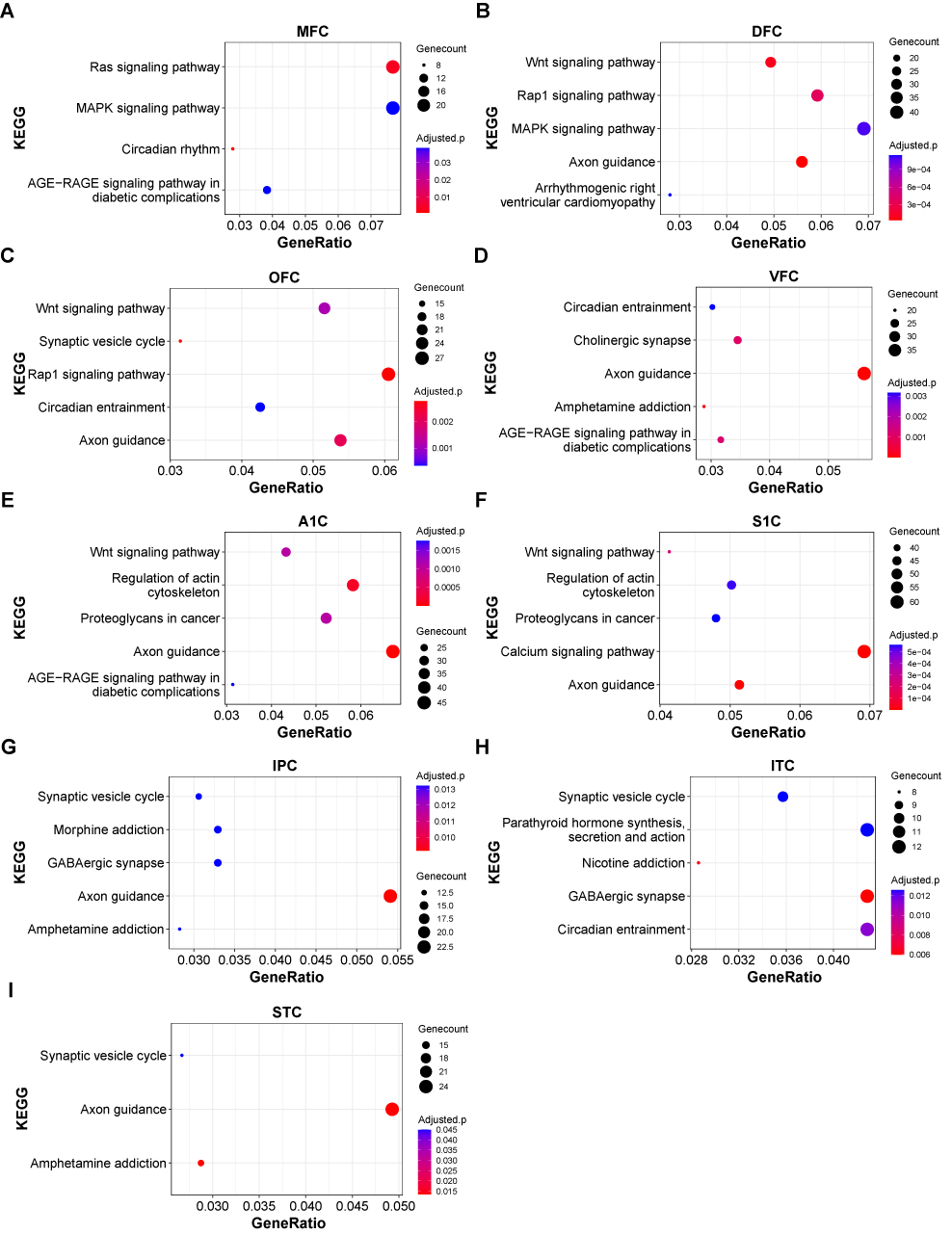

Supplement: Supplementary Figure 4 — Kegg pathways of DEGs in different brain regions in rhesus macaques. (A–I) represent top enriched functional categories in the developmental DEGs of Mfc, Dfc, Ofc, Vfc, A1C, S1C, Ipc, Itc, and Stc. Pathway terms were sorted from smallest to largest by p-value. Smaller p-values are indicated by darker red color. The size of the circle represents the number DEGs enriched and the x-axis represents the odd ratio. [file Image_4.TIF]
